# Conceptual DFT, QTAIM, and Molecular Docking Approaches to Characterize the T-Type Calcium Channel Blocker Anandamide

**DOI:** 10.3389/fchem.2022.920661

**Published:** 2022-07-14

**Authors:** Maricruz Rangel-Galván, María Eugenia Castro, Jose Manuel Perez-Aguilar, Norma A. Caballero, Francisco J. Melendez

**Affiliations:** ^1^ Lab. de Química Teórica, Centro de Investigación, Depto. de Fisicoquímica, Facultad de Ciencias Químicas, Benemérita Universidad Autónoma de Puebla, Puebla, Mexico; ^2^ Centro de Química, Instituto de Ciencias, Benemérita Universidad Autónoma de Puebla, Puebla, Mexico; ^3^ Facultad de Ciencias Biológicas, Benemérita Universidad Autónoma de Puebla, Puebla, Mexico

**Keywords:** anandamide, conceptual DFT, QTAIM, molecular docking, chemical reactivity, pain and depression

## Abstract

The anandamide is a relevant ligand due to its capacity of interacting with several proteins, including the T-type calcium channels, which play an important role in neuropathic pain and depression disorders. Hence, a detailed characterization of the chemical properties and conformational stability of anandamide may provide valuable information to understand its behavior in a biological context. Herein, conceptual DFT and QTAIM analyses were performed to theoretically characterize the chemical reactivity properties and the structural stability of conformations of anandamide, using the BP86/cc-pVTZ level of theory. Global reactivity description, based on conceptual DFT, indicates that the hardness increases and the electrophilicity index decreases for both, the hairpin and U-shape conformers relative to the extended conformers. Also, an increase in the chemical potential value and a decrease in the electronegativity and the electrophilicity index is observed in the ethanolamide open ring conformers in comparison with the corresponding closed ring structures. In addition, regarding the characterization of local reactivity descriptors, the maximum values of the Fukui and Parr functions indicate that the most probable location for a nucleophilic attack is either the hydroxyl oxygen located in the ethanolamide closed ring conformers or the carbonyl oxygen present in the open ring conformers. The most probable location for an electrophilic attack is in the alkyl double bond region in all anandamide conformers. According to the QTAIM results, the intramolecular hydrogen bond formation stabilizing the structure of anandamide has interaction energy values for the closed ring conformations of 12.33–12.46 kcal mol^−1^, indicating a strong interaction. Lastly, molecular docking calculations determined that a region in the pore, denominate as pore-blocking, is a probable site for the interaction of anandamide with the human Ca_v_3.2 isoform of the T-type calcium channel family. The pore-blocking site contains hydrophobic residues where the non-polar part in the final alkyl region of anandamide established mainly alkyl-alkyl interactions, while the polar part (the ethanolamide group) interacts with the polar residue S900. The information based on conceptual DFT presented may aid in the design of drugs with similar chemical characteristics as those identified in anandamide so as to bind anandamide-interacting proteins, including the T-type calcium channels.

## Graphical Abstract

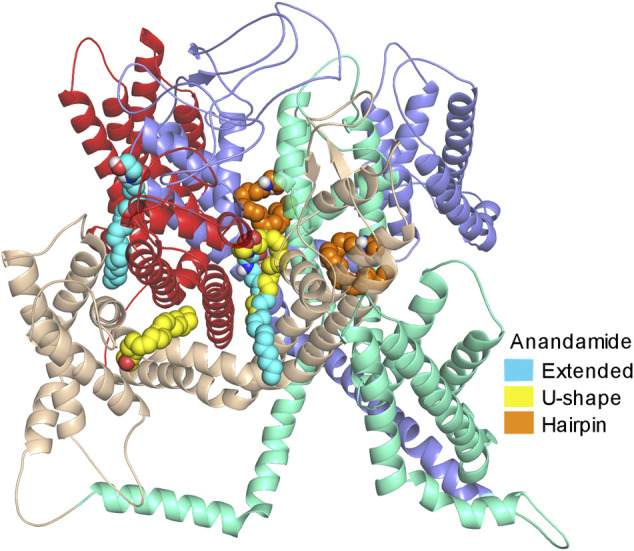

## Introduction

Anandamide is a fatty acid neurotransmitter that is synthesized enzymatically in the brain from arachidonic acid (AA) and ethanolamide substrates (Devane and Axelrod, 1994); however, other synthetic pathways involving the *N*-arachidonoyl phosphatidylethanolamine (PE) precursor and the phosphodiesterase enzyme, have been also proposed (Sugiura et al., 2002). Although anandamide is an endogenous endocannabinoid ligand known to bind mainly to the cannabinoid receptors, it is also capable of directly modulating a variety of ion channels, such as potassium channels, sodium channels, TRPV1 (Transient Receptor Potential Vanilloid type 1), and the T-type calcium channels (Van Der Stelt and Di Marzo, 2005; Oz, 2006). Along these lines, it has been proved that blocking both, the T-type calcium channel and the cannabinoid receptors is an important therapeutic strategy for treating neuropathic and inflammatory pain (You et al., 2011; Gadotti et al., 2013; Bladen et al., 2015). Moreover, the T-type calcium channels and the CB1/CB2 cannabinoid receptors are involved in disorders associated with neuropathic pain, mental pain, suicidal behavior, and depression (Pacher et al., 2006; Todorovic and Jevtovic-Todorovic, 2013; Tibbs et al., 2016; Colino et al., 2018; Snutch and Zamponi, 2018; Yang et al., 2018). Among the three isoforms that constitute the T-type calcium channels (Ca_v_3.1, Ca_v_3.2, and Ca_v_3.3), the Ca_v_3.2 contributes significantly to the nociceptive pathway which is responsible for the physiological and pathological mechanism of pain transmission. It was demonstrated by electrophysiological experiments that anandamide directly inhibits the T-type calcium channels during neural activities with a preference for the Ca_v_3.2 isoform displaying IC_50_ values for the Ca_v_3.1, Ca_v_3.2, and Ca_v_3.3 channels of 4.15 µM, 330 nM, and 1.10 µM, respectively (Chemin et al., 2001). Using electrophysiology recordings, it was found that the hydroxyl group and the alkyl chain from anandamide play important roles in the inhibition mechanism of the T-type calcium channel by anandamide. In detail, it was found that the chain length and the presence, position, and all-*cis* isomerism of the alkyl chain double bonds, are all important factors for the degree of anandamide inhibition. Interestingly, the inhibition can occur even though the alkyl chain contains either 18 or 22 carbons instead of 20 carbons. Also, it was observed that a decrease in the degree of unsaturation in anandamide reduces its blocking effect, with a fully saturated alkyl chain displaying negligible inhibitory effect. The inhibition increases when double bonds are near the carboxyl group and have no effect when a *trans*-configuration and triple bonds are considered (Chemin et al., 2007).

Experimental X-ray structures found the alternated form of anandamide (*cis-trans-cis-trans*) and all-*trans* anandamide in interaction with its intracellular transporter, the fatty acid binding protein FABP5 in mouse (mFABP5) and human (hFABP5), respectively (Sanson et al., 2014). Also, both isomer molecules the synthesized all-*trans* anandamide and the natural *cis*-anandamide are equally good substrates for fatty acid amide hydrolase FAAH enzyme (Ferreri et al., 2008). Nevertheless, it has been observed that endogenous *trans* and *cis-trans* of AA isomerization originate from diet and by the action of the nitrogen dioxide radicals (NO_2_) (Zghibeh et al., 2004). This could indicate that the alternated (*cis-trans-cis-trans*) and all-*trans* anandamide forms may be present in certain conditions but the most relevant form during receptor interaction is the all-*cis* anandamide.

Regarding the interaction of anandamide with the cannabinoid receptors, a mutagenesis study demonstrated the importance of the amino acid F3.25 (following B&W nomenclature) for binding anandamide in the binding site of the CB1 receptor while a computational docking study showed interactions between anandamide, in a hairpin conformation, with the same receptor (McAllister et al., 2003). In a crystallographic study where the structure of the CB1 receptor was solved, the authors used the X-ray structure to dock various agonist molecules, including anandamide and found that the molecule prefers a hairpin conformation in the binding site of the receptor (Hua et al., 2016). Although there is significant information regarding the interaction of anandamide and various proteins, there is no information regarding the interaction of this important compound with the T-type calcium channels. Herein we used computational methods to characterize the reactivity of anandamide in the ligand-receptor context, evaluate its structural stability, and study the interaction of anandamide with the structure of the Ca_v_3.2 channel. Conceptual DFT is used for the chemical reactivity interpretation by global and local reactivity descriptors, which are used to identify the possible interacting regions of anandamide at the Ca_v_3.2 channel. QTAIM analyses were performed to theoretically characterize the structural stability of the preferred conformations of anandamide (Rangel-Galván et al., 2022a). These calculations were made using the BP86/cc-pVTZ level of theory. Finally, molecular docking calculations are used to characterize the main amino acids interacting in the Ca_v_3.2/anandamide complex.

## Computational Methods

### Conceptual DFT Analysis

The conceptual DFT approach (Domingo et al., 2016; Frau and Glossman-Mitnik, 2017; Frau and Glossman-Mitnik, 2018) was used to evaluate the global reactivity descriptors, such as chemical potential (μ), electronegativity (χ), hardness (η), softness (s), and electrophilicity index (ω), from the Frontier molecular orbitals energies, E_HOMO_ and E_LUMO_, using the following equations: μ = (E_HOMO_ + E_LUMO_) ⁄ 2; χ = −(E_HOMO_ + E_LUMO_) ⁄ 2; η = E_LUMO_−E_HOMO_; s = 1 ⁄ η, ω = μ^2^ ⁄ 2η. In addition, the local reactivity descriptors, such as the Fukui functions f(r)  for electrophilic $f^{-}\left(r\right)$ and nucleophilic $f^{+}\left(r\right)$ attacks, and the dual descriptor $f^{\left(2\right)}\left(r\right)$ was calculated using the following equations: $f_{k}^{-}=q_{k}\left(N\right)-q_{k}\left(N-1\right)$ ; $f_{k}^{+}=q_{k}\left(N+1\right)-q_{k}\left(N\right)$ ; $f_{k}^{\left(2\right)}=f_{k}^{+}-f_{k}^{-}$ , where $q_{k}$ are the electronic population of the *k*th atom of the molecule with N−1 , N , and N+1 electrons (Yang and Mortier, 1986; Morell et al., 2005). Hirshfeld population analysis was carried out by using the Multiwfn program (Lu and Chen, 2012). The Parr functions P(r) for electrophilic $P^{-}\left(r\right)$ and nucleophilic $P^{+}\left(r\right)$ attacks were performed using the equations: $P_{k}^{-}=\rho_{k}^{\mathrm{rc}}$ ; $P_{k}^{+}=\rho_{k}^{\mathrm{ra}}$ , where $\rho_{k}^{\mathrm{rc}}\;$ and $\rho_{k}^{\mathrm{ra}}$ is the atom spin density (ASD) of the *k*th atom at the cation and the anion of the molecule, respectively (Chamorro et al., 2013; Domingo et al., 2013). The data of the optimized conformers of anandamide were obtained from the BP86 functional (Becke, 1988) and cc-pVTZ basis set (Dunning, 1989) in chloroform solvent, as previously reported (Rangel-Galván et al., 2022a). DFT calculations have been shown to be a reliable tool to analyze reactivity descriptors within the framework of conceptual DFT (De Proft et al., 2003; Vivas-Reyes et al., 2003; Vivas-Reyes et al., 2008). Calculations were carried out using the Gaussian16 program (Frisch et al., 2016).

### QTAIM Analysis

Topological parameters, based on the QTAIM approach (Matta and Boyd, 2007; Hernández-Paredes et al., 2017; Bayat and Fattahi, 2019), were calculated, such as electronic density, ρ(r) , the Laplacian, $\nabla^{2}\rho \left(r\right)$ , the Lagrangian kinetic energy, *G*, the Hamiltonian kinetic energy, *H*, the potential energy density, *V*, the interaction energy, *E*_*H … Y*_, the interatomic distance *D*_*inter*_, and the delocalization indexes, *DI*, in order to characterize the hydrogen bonds and other intramolecular interactions in the anandamide conformers. The intramolecular hydrogen bond in the ethanolamide (EA) group was evaluated in the anandamide conformers with the closed ring. These topological properties were calculated using the equations: H(r)=G(r)−V(r) , and $E_{H\cdots Y}=\frac{1}{2}V\left(r\right)$ . The QTAIM calculations were performed using the AIMAll (Version 17.11.14) program (Tood, 2017).

### Molecular Docking Analysis

Molecular docking calculations were carried out for the Ca_v_3.2 calcium channel and the conformers of anandamide. The rigid blind docking was performed using the AutoDockVina (version 1.1.2) program (Trott and Olson, 2010). AutoDockVina uses a genetic algorithm as a searching method and the binding free energy parameter (−ΔG) as the scoring function. The binding free energy is calculated through the equation: $c=\sum_{i<j}f_{t_{i}t_{j}}\left(r_{ij}\right)$ , where each atom i is assigned an atom type $t_{i}$ , and $f_{t_{i}t_{j}}$ are a symmetric set of interaction functions occurring at the interatomic distance $r_{ij}$ . The sum can be seen as the addition of the intermolecular and intramolecular contributions of all the pairs of atoms interacting with each other. The optimization algorithm finds the global minimum c in terms of the free energy values obtained for each conformation (Trott and Olson, 2010). The grid dimension space was 90 Å × 110 Å × 110 Å, containing the entire receptor structure. The default exhaustiveness value of 8 and 80 were used with 10 resulting poses. The structures of anandamide conformers, used as ligands, were obtained from the optimized structures at the BP86/cc-pVTZ level of theory in chloroform solvent (Rangel-Galván et al., 2022a). The Ca_v_3.2 structure, used as the receptor, was taken from the homology model previously reported (Rangel-Galván et al., 2022b). The ligand Gasteiger charges were calculated and hydrogen atoms were added with Autodock tools (Sanner, 1999). The interactions were visualized with PyMOL v2.0 (Schrödinger, 2017).

## Results and Discussion

### Conceptual DFT Analysis

Six minimum energy structures for anandamide were selected for the conceptual DFT analysis: extended with closed EA ring (E_closed_), extended with open EA ring (E_open_), U-shape with closed EA ring (U_closed_), U-shape with open EA ring (U_open_), hairpin with closed EA ring (H_closed_), and hairpin with open EA ring (H_open_). Global reactivity descriptors for the six conformers of anandamide E_closed,_ E_open_, U_closed_, U_open_, H_closed_, and H_open_, were evaluated: chemical potential (μ), electronegativity (χ), hardness (η), softness (s), and electrophilicity index (ω), according to conceptual DFT approach (Domingo et al., 2016; Frau and Glossman-Mitnik, 2017). Table 1 summarized the HOMO and LUMO energies and the global reactivity descriptors for the conformers of anandamide.

**TABLE 1:** HOMO and LUMO energies and global reactivity descriptors (eV) of the conformers of anandamide obtained at the BP86/cc-pVTZ level of theory in chloroform.

	E_closed_	E_open_	U_closed_	U_open_	H_closed_	H_open_
^a^LUMO	−1.32	−1.29	−1.24	−1.21	−1.09	−1.03
^a^HOMO	−5.67	−5.72	−5.87	−5.80	−5.85	−5.75
µ	−3.54	−3.50	−3.56	−3.50	−3.47	−3.39
χ	3.54	3.50	3.56	3.50	3.47	3.39
η	4.43	4.43	4.63	4.59	4.76	4.71
S	0.23	0.23	0.22	0.22	0.21	0.21
ω	1.41	1.38	1.37	1.33	1.26	1.22

a From Ref. (Rangel-Galván et al., 2022a).

For the E_closed_/E_open,_ U_closed_/U_open_, and H_closed_/H_open_ shapes of anandamide, in Table 1 it is observed that there are no significant changes when anandamide changes the curvature of this structure. Some slight differences are found in hardness, softness, and the electrophilicity index. The hardness increases a 4.6/3.5 and 7.4/6.3% for U_closed_/U_open_ and H_closed_/H_open_ shapes, respectively, relative to the E_closed_/E_open_ conformers. The softness suffers a decrement keeping the same proportion. Finally, a decrease of the electrophilicity index of 3.3/3.6 and 10.6/12.0% is observed for U_closed_/U_open_ and H_closed_/H_open_ shapes, respectively, with respect to the E_closed_/E_open_ conformers. The effect of the ethanolamide (EA) open ring conformers in comparison with the corresponding closed shapes is seen in slight differences, an increase in chemical potential, and a decrease in electronegativity and electrophilicity index. For example, for the electrophilicity index, the decrease represents a 2, 2.3, and 3.5% for E_open_, U_open,_ and H_open_ with respect to their corresponding closed shapes.

In addition to anandamide as a dual ligand for the T-type calcium channels and the cannabinoid receptors, the NMP compounds have been shown to be capable of binding both proteins (You et al., 2011; Gadotti et al., 2013; Bladen et al., 2015), which is relevant in the treatment of pain disorders.

Taking into account the conceptual DFT analysis previously executed for the NMP compounds, the global reactivity descriptor that changes the most when anandamide and NMP compounds are compared (NMP-7 and H_closed_ shape for representation), is the hardness of 51%, from 4.76 eV in the H_closed_ conformation of anandamide and 3.14 eV in the NMP-7 ligand (Rangel-Galván et al., 2022b). Anandamide has a higher resistance to changing electronic distribution compared to the NMP compounds (Rangel-Galván et al., 2022b). It is worth mentioning that significant changes in the hardness index have been observed when a ligand-receptor interaction process is modeled (Aliste, 2000). The electrophilicity index is another uneven descriptor; a higher value corresponds to the NMP compounds with respect to anandamide conformers with a difference of 37.6%. The electrophilicity index results are well correlated to the receptor affinity properties and biological activity (Parthasarathi et al., 2004).

**FIGURE 1:**
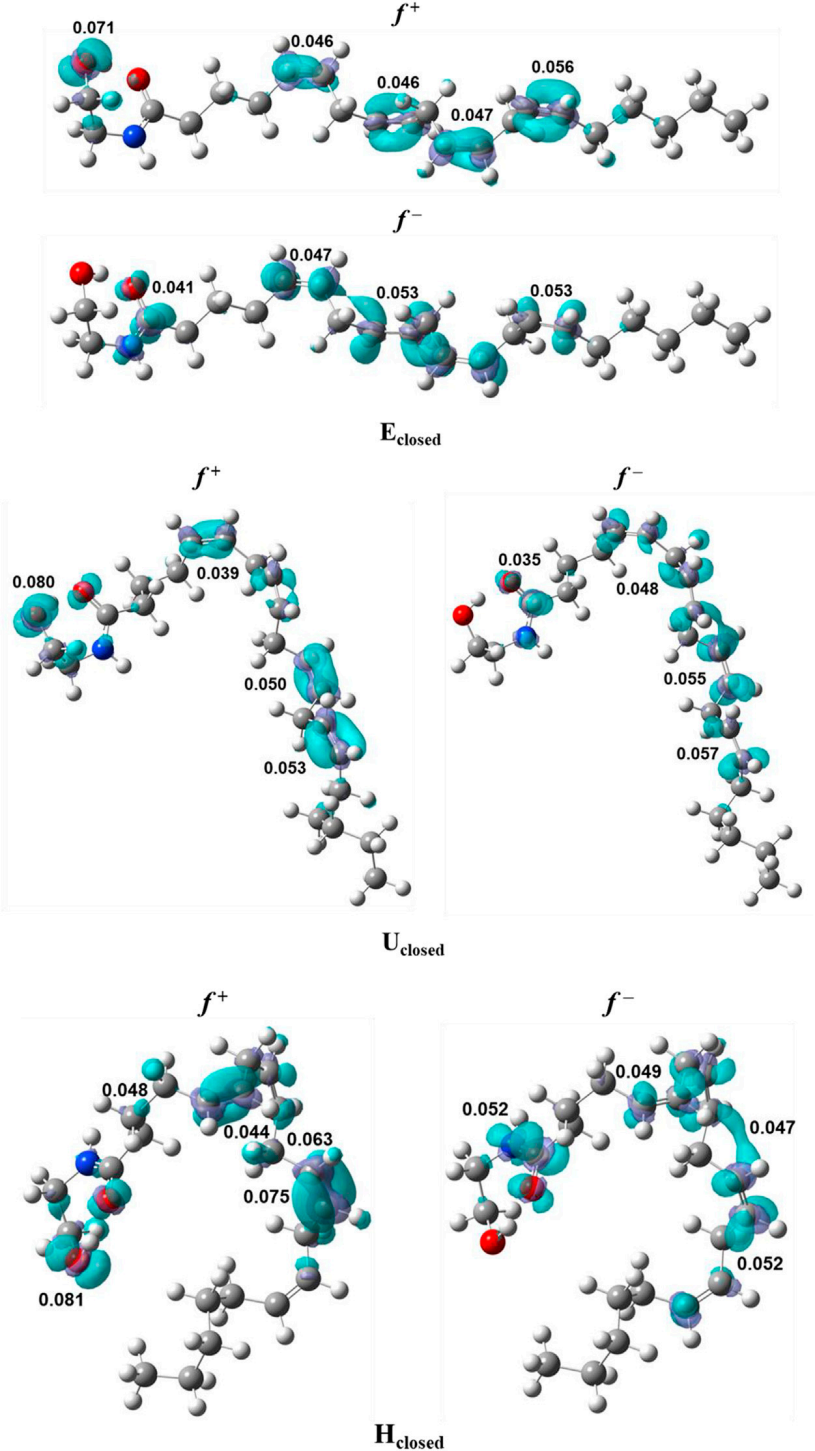
Fukui functions, *f* ^*+*^ and *f* ^*–*^, of the conformers of anandamide: E_closed_, U_closed_, and H_closed_, obtained at the BP86/cc-pVTZ level of theory in chloroform.

Respect the local reactivity descriptors, Table 2 shows the condensed Fukui functions, $f^{+}\left(r\right)$ and $f^{-}\left(r\right)$ , the dual descriptor, $f^{\left(2\right)}\left(r\right)$ , and the Parr functions, $P^{-}\left(r\right)$ and $P^{+}\left(r\right)$ , the conformers of anandamide with a closed ring of ethanolamide (EA). For E_closed_, U_closed_, and H_closed_ shapes of anandamide, values of Fukui functions were found in the ranges *f* ^*+*^ = 0.007–0.071 and *f* ^*–*^ = 0.007–0.053 for E_closed_, *f* ^*+*^ = 0.012–0.080 and *f* ^−^ = 0.008–0.057 for U_closed_, and *f* ^*+*^ = 0.005–0.081 and *f* ^*–*^ = 0.009–0.052 for H_closed_. In addition, for open ring the values were found in the ranges *f* ^*+*^ = 0.020–0.079 and *f* ^*–*^ = 0.022–0.059 for E_open_, *f* ^*+*^ = 0.023–0.087 and *f* ^*–*^ = 0.017–0.064 for U_open_, and *f* ^*+*^ = 0.022–0.089 and *f* ^*–*^ = 0.028–0.060 for H_open_, as shown in Supplementary Table S1. The most relevant values are in the atom O22 of the hydroxyl group in the closed ring conformers, O21 of the carbonyl group in the open ring conformers in the ethanolamide (EA) group for nucleophilic attacks, and C12 in the middle alkyl region (Alkyl-M) for electrophilic attack for all the conformers. For the Parr functions, it is found a correspondence in the location of the highest values with the values found in the Fukui indices. Figure 1 presents the isosurfaces for Fukui functions with the major value for *f* ^*+*^ and *f* ^*–*^ for all shapes of anandamide. In Figure 1, it is observed that *f* ^*+*^ distributions are located on the hydroxyl oxygen O22 in closed conformers, and in the carbonyl oxygen O21 in open conformers, as shown in Supplementary Figure S1 in the SM section, also in the vinylic carbons on the Alkyl-M region for all conformers. While the *f* ^*–*^ distribution is located on the carbonyl carbon C2 in the closed conformers, it is also found on the vinylic carbons in the Alkyl-M region for all conformers. This finding is in accord with the experimental data which show that the double bonds region for anandamide is an essential factor for the T-type calcium channel blocking mechanism (Chemin et al., 2014).

**TABLE 2:** Fukui functions, $f^{+}\left(r\right)$ and $f^{-}\left(r\right)$ , dual descriptor, $f^{\left(2\right)}\left(r\right)$ , and Parr functions, $P^{-}\left(r\right)$ and $P^{+}\left(r\right)$ the conformers of anandamide: E_closed_, U_closed_, and H_closed_, obtained at the BP86/cc-pVTZ level of theory in chloroform.

		$f^{+}\left(r\right)$	$f^{-}\left(r\right)$	$f^{\left(2\right)}\left(r\right)$	$P^{-}\left(r\right)$	$P^{+}\left(r\right)$
E_closed_	C1	0.007	0.041	0.034	0.002	0.088
	C5	0.046	0.047	0.000	0.075	0.071
	C6	0.038	0.042	0.004	0.063	0.070
	C8	0.046	0.053	0.007	0.074	0.084
	C9	0.043	0.052	0.008	0.069	0.084
	C11	0.045	0.048	0.003	0.072	0.080
	C12	0.047	0.053	0.006	0.074	0.083
	C14	0.048	0.029	0.018	0.084	0.049
	C15	0.056	0.039	0.017	0.091	0.060
	O22	0.071	0.007	0.064	0.117	0.001
U_closed_	C1	0.012	0.039	0.027	0.007	0.084
	C5	0.038	0.039	0.001	0.061	0.057
	C6	0.039	0.037	0.002	0.070	0.068
	C8	0.034	0.048	0.014	0.052	0.077
	C9	0.030	0.044	0.015	0.048	0.068
	C11	0.050	0.055	0.005	0.082	0.092
	C12	0.049	0.057	0.008	0.081	0.089
	C14	0.047	0.036	0.011	0.087	0.063
	C15	0.053	0.044	0.009	0.084	0.067
	O22	0.080	0.008	0.072	0.127	0.001
H_closed_	C1	0.013	0.052	0.039	0.006	0.114
	C5	0.048	0.034	0.014	0.059	0.040
	C6	0.044	0.034	0.010	0.058	0.077
	C8	0.039	0.049	0.010	0.083	0.076
	C9	0.028	0.043	0.015	0.082	0.071
	C11	0.063	0.047	0.017	0.046	0.077
	C12	0.075	0.052	0.023	0.044	0.082
	C14	0.005	0.027	0.022	0.101	0.043
	C15	0.013	0.035	0.022	0.089	0.053
	O22	0.081	0.009	0.072	0.114	0.001

### QTAIM Analysis

**FIGURE 2:**
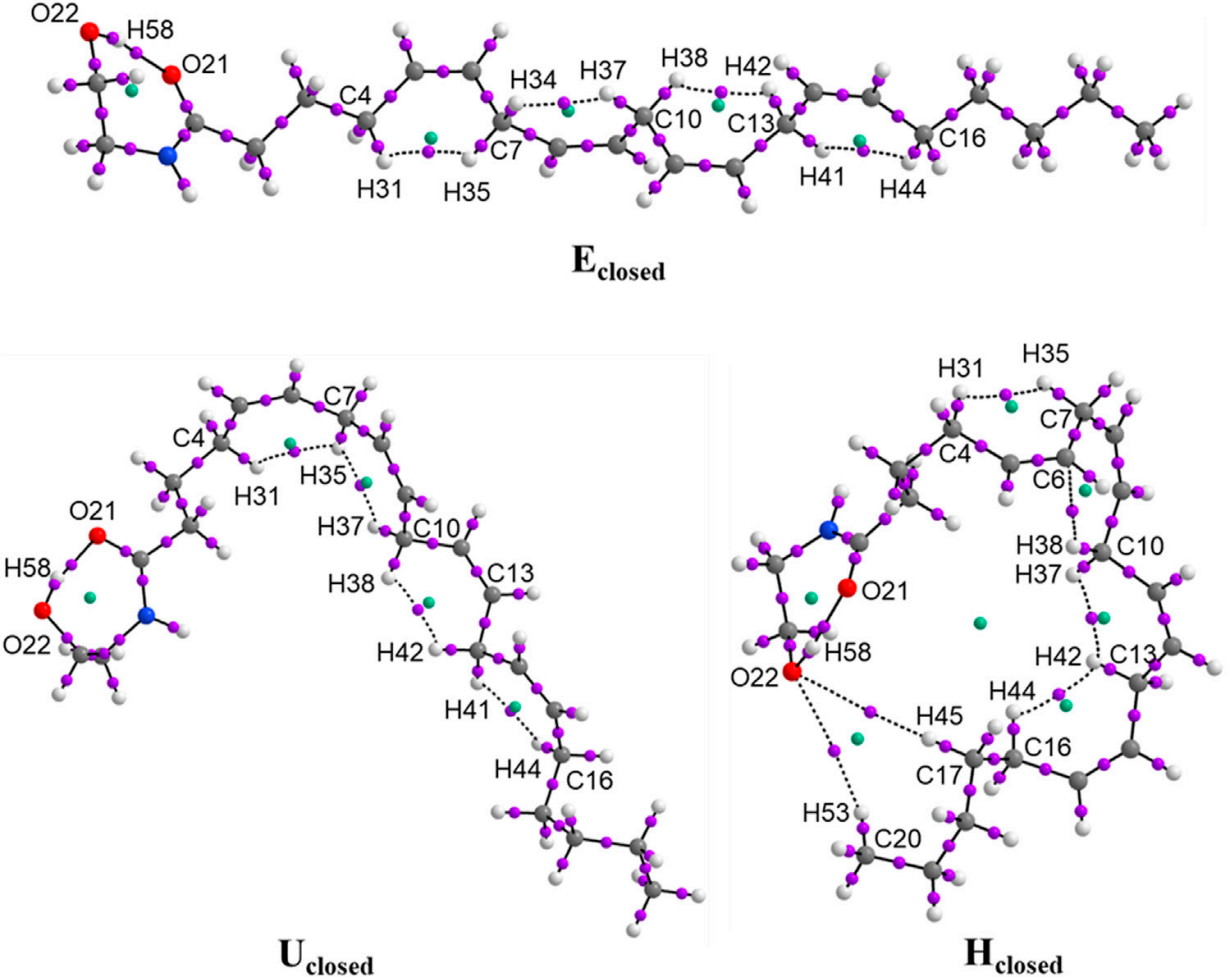
Molecular graphs of the conformers of anandamide: E_closed_, U_closed_, and H_closed_, obtained at the BP86/cc-pVTZ level of theory in chloroform.

The QTAIM analysis was focused to characterize the intramolecular hydrogen bond of the closed ring formed in the ethanolamide group in the conformers E_closed_, U_closed_, and H_closed_ of anandamide. This interaction of the hydrogen bond generates a ring structure of seven atoms in the EA group. The stability of the conformers with a closed ring of anandamide by the hydrogen bond formation effect is analyzed by means of interaction energy for each closed ring anandamide conformer from electronic density obtained from the BP86/cc-pVTZ level of theoretical calculations in chloroform. Table 3 and Supplementary Table S2 show the values of electronic density, ρ(r) , the Laplacian, $\nabla^{2}\rho \left(r\right)$ , the Lagrangian kinetic energy, *G*, the Hamiltonian kinetic energy, *H*, the potential energy density, *V*, the interaction energy, *E*_*H … Y*_, the interatomic distance, *D*_*inter*_, and the delocalization indexes, *DI*, of the main bond critical points (BCP) of the anandamide conformers. Atom labels correspond to Figure 2.

**TABLE 3:** Topological parameters (a.u.), *E*_*H … Y*_ (kcal mol^−1^), and interatomic distances (D_inter_, Å) of the conformers of anandamide: E_closed_, U_closed_, and H_closed_, obtained at the BP86/cc-pVTZ level of theory in chloroform.

BCP	ρ(r)	∇^2^ρ	G (r)	V (r)	H (r)	E_H … Y_	D_inter_	DI	RCP
	**E_closed_**								
O_22_H_58_−O_21_	0.0430	0.0959	0.0317	−0.0393	−0.0076	12.33	1.75846	0.1106	0.0115
C_4_H_31_−H_35_C_7_	0.0118	0.038	0.0079	−0.0063	0.0016	1.98	2.06071	0.0284	0.0113
C_7_H_34_−H_37_C_10_	0.0120	0.0386	0.0081	−0.0065	0.0016	2.04	2.04591	0.0289	0.0115
C_10_H_38_−H_42_C_13_	0.0121	0.0386	0.0081	−0.0065	0.0016	2.04	2.04497	0.0289	0.0115
C_13_H_41_−H_44_C_16_	0.0118	0.0380	0.0079	−0.0063	0.0016	1.98	2.06096	0.0284	0.0113
	**U**_**closed**_								
O_22_H_58_−O_21_	0.0433	0.0961	0.0319	−0.0397	−0.0078	12.46	1.75571	0.1110	0.0115
C_4_H_31_−H_35_C_7_	0.0112	0.0367	0.0076	−0.006	0.0016	1.88	2.08868	0.0263	0.0100
C_7_H_35_−H_37_C_10_	0.0116	0.0378	0.0079	−0.0063	0.0016	1.98	2.06339	0.0273	0.0112
C_10_H_38_−H_42_C_13_	0.0121	0.0384	0.0080	−0.0065	0.0015	2.04	2.04242	0.0294	0.0115
C_13_H_41_−H_44_C_16_	0.0119	0.0387	0.0081	−0.0065	0.0016	2.04	2.05663	0.0284	0.0115
	**H**_**closed**_								
O_22_H_58_−O_21_	0.0431	0.0953	0.0317	−0.0395	−0.0078	12.39	1.75686	0.1109	0.0116
C_4_H_31_−H_35_C_7_	0.0120	0.0382	0.008	−0.0064	0.0016	2.01	2.04967	0.0290	0.0114
C_6_−H_38_C_10_	0.0135	0.0395	0.0087	−0.0075	0.0012	2.35	2.2993	0.0449	0.0092
C_10_H_37_−H_42_C_13_	0.0115	0.0374	0.0078	−0.0062	0.0016	1.95	2.07088	0.0270	0.0110
C_13_H_42_−H_44_C_16_	0.0114	0.0372	0.0077	−0.0061	0.0016	1.91	2.07819	0.0269	0.0110
C_17_H_45_−O_22_	0.0016	0.0050	0.0009	−0.0006	0.0003	0.19	3.40010	0.0100	0.0013
C_20_H_53_−O_22_	0.0015	0.0050	0.0009	−0.0006	0.0003	0.19	3.43546	0.0095	0.0013

From the results, it is observed the non-covalent interaction corresponds to the hydrogen bond in the EA group in O21⋯H58-O22 with ρ(**r**) = 0.043 and ∇^2^ρ(r) = ∼ 0.096 a.u. for conformers E_closed_, U_closed_, and H_closed_. These values are in agreement with those reported in the literature of 0.002–0.035 and 0.024–0.139 a.u., respectively (Popelier, 2000). On the other hand, the sign of H(**r**) parameter indicates, in the three closed conformers, that the accumulation of charge density on the hydrogen bond is a stabilizing bond (H(r) < 0) (Cremer and Kraka, 1984). In addition, the DI parameters have values in the range of 0.1106–0.1110 a.u. for the intramolecular hydrogen bond O21⋯H58−O22 in the three closed conformers, indicating that is an interaction <1.0 corresponding to a non-covalent interaction, while the DI for BCP between H58−O22 have values in the range of 0.5371–0.5402 a.u., indicating that this bond has a minor strength than a single bond (value close to 1.0 a.u.), due to the H58 is also contributing to the hydrogen bond formation with the atom O21. The interaction energy of the intramolecular hydrogen bond O21⋯H58−O22 corresponds to 12.33–12.46 kcal mol^−1^ and it forms an RCP with ρ(r) = 0.0115–0.0116 a.u. for the three conformers, being slightly major the *E*_*H … Y*_ value in the conformer U_closed_. It indicates that this hydrogen bond has a stabilizing effect larger than in the other conformers with the closed ring. The value of ρ(**r**) in the RCP of ethanolamide indicates that the ring formation stabilizes in the same order as the structures in the three conformers of the closed ring.

The non-covalent interactions H⋯H in the Alkyl-M region with a value of ρ(**r**) in the range of 0.011–0.020 a.u. generate the formation of four ring structures with ρ(**r**) in the RCP of 0.010–0.015 a.u. for the three closed conformers. The interaction energies between H⋯H are very small indicating weak interactions with values of DI of 0.03 a.u. approximately. Only in the conformer H_closed_, is observed the non-covalent interaction C_6_−H_37_C_10_ with values of ρ(r) in the BCP and RCP of 0.0135 and 0.0092 a.u., respectively, and a DI value of 0.045 a.u. In addition, the conformer H_closed_ forms an additional ring structure for the interactions between C_17_H_45_−O_24_ and C_20_H_53_−O_24_ with the value of ρ(**r**) = 0.0013 a.u. in the RCP. On the other hand, the DI values between atoms C5 = C6, C8 = C9, C11 = C12, and C14 = C15 in the Alkyl-M region are 1.77 a.u., corroborating the *cis* double bonds formation in the three closed conformers. Figure 2 and Supplementary Figure S2 show the molecular graphs of the anandamide conformers. The purple dots represent the bond critical points (BCP) and the green points represent the ring critical points (RCP).

### Molecular Docking Analysis

**FIGURE 3:**
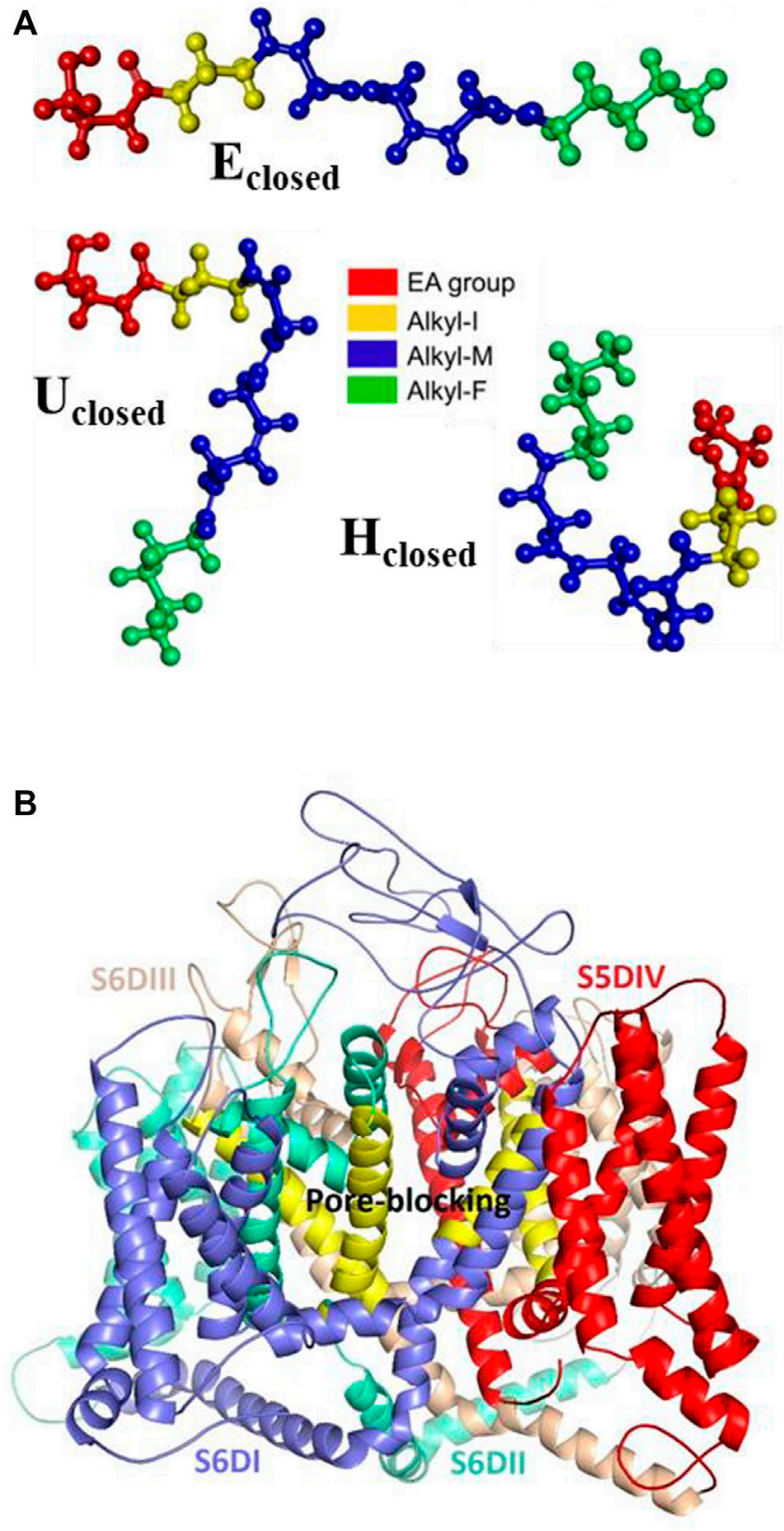
Molecular structures. **(A)** Anandamide conformers E_closed_, U_closed_, and H_closed_, and **(B)** Ca_v_3.2 channel showing the main interacting segments.

Given the importance of mixed T-type/cannabinoid blockage in the pain diseases context, in this molecular docking analysis, and as a comparison, we consider the five proposed sites of the complex formed by the Ca_v_3.2 channel and other dual blockers, the NMP compounds. The sites named S6DI, S6DII, S6DIII, S5DIV, and pore-blocking (Figure 3), are located in the transmembrane region, where aromatic and hydrophobic amino acids are prevalent (Rangel-Galván et al., 2022b). In this work, were analyzed the conformers E_closed_, U_closed_, and H_closed_ in interaction with the Ca_v_3.2 calcium channel. In addition, the conformers E_open_, U_open,_ and H_open_ were included to confirm our previous observations that the closed ring structures are more stable than their open counterparts (Rangel-Galván et al., 2022a) and probably more like to participate in the interaction with biological macromolecules, specifically with the Ca_v_3.2 calcium channel. Figure 3 shows the anandamide conformers as ligands and the Ca_v_3.2 channel as the receptor, showing the main interacting segments.

**FIGURE 4:**
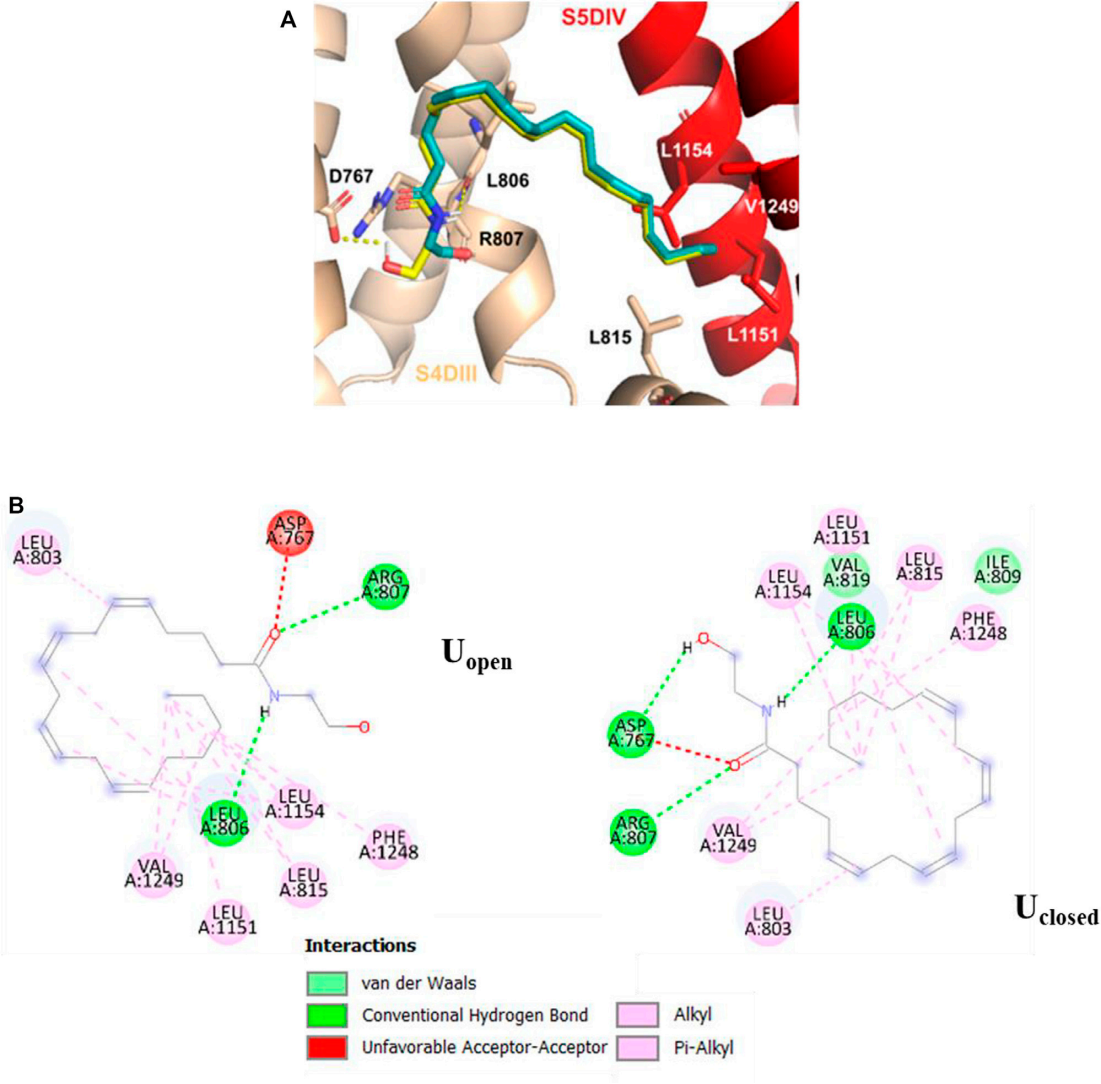
Molecular interactions of U_open_ (cyan) and U_closed_ (yellow) anandamide conformers with Ca_v_3.2. **(A)** 3D perspective of S5DIV site, U_closed_ and U_open_ conformers forming two hydrogen bonds with residues L806 and R807, U_closed_ additionally forms a hydrogen bond with D767 **(B)** 2D perspective interaction for each anandamide conformer in the S5DIV site.

In the S5DIV site (Figure 4), the U_closed_ and U_open_ conformers place their polar head groups into the S4DIII segment, where the polar interactions are formed with the hydrogen N23−H⋯O of U_closed_ and the carbonyl oxygen of L806 (backbone), and the hydrogen O22−H⋯O of U_open_ and the carbonyl oxygen of R807 (both residues are located at 2.6 Å from the ligands). Also, is observed an unfavorable acceptor-acceptor interaction with O21 of U_closed_ and U_open_ conformers and the side chain carbonyl oxygen of D767 residue (Figure 4). The U_closed_ conformer forms an additional hydrogen bond, formed by hydrogen O22−H⋯O and the side chain carbonyl oxygen of acidic residue D767, however, this interaction does not contribute to the affinity of the ligand for the calcium channel with interaction energy of −9.2 kcal mol^−1^ for both conformers. The non-polar portion of both conformers (U_closed_ and U_open_) is placed in the S5DIV segment where the hydrophobic residues are located forming alkyl–alkyl interactions with the L815, L1151, L1154, and V1249 residues. In the X-ray structures of the TRPV5, TRPV2, and TRPV1 channels, which are close members of the T-type calcium channels, in complex with econazole (Hughes et al., 2018), resiniferatoxin (Zubcevic et al., 2018), and capsazepine (Gao et al., 2016), respectively, we observed ligand binding sites located in equivalent positions to that defined as the segment S5DIV in the Ca_v_3.2.

**FIGURE 5:**
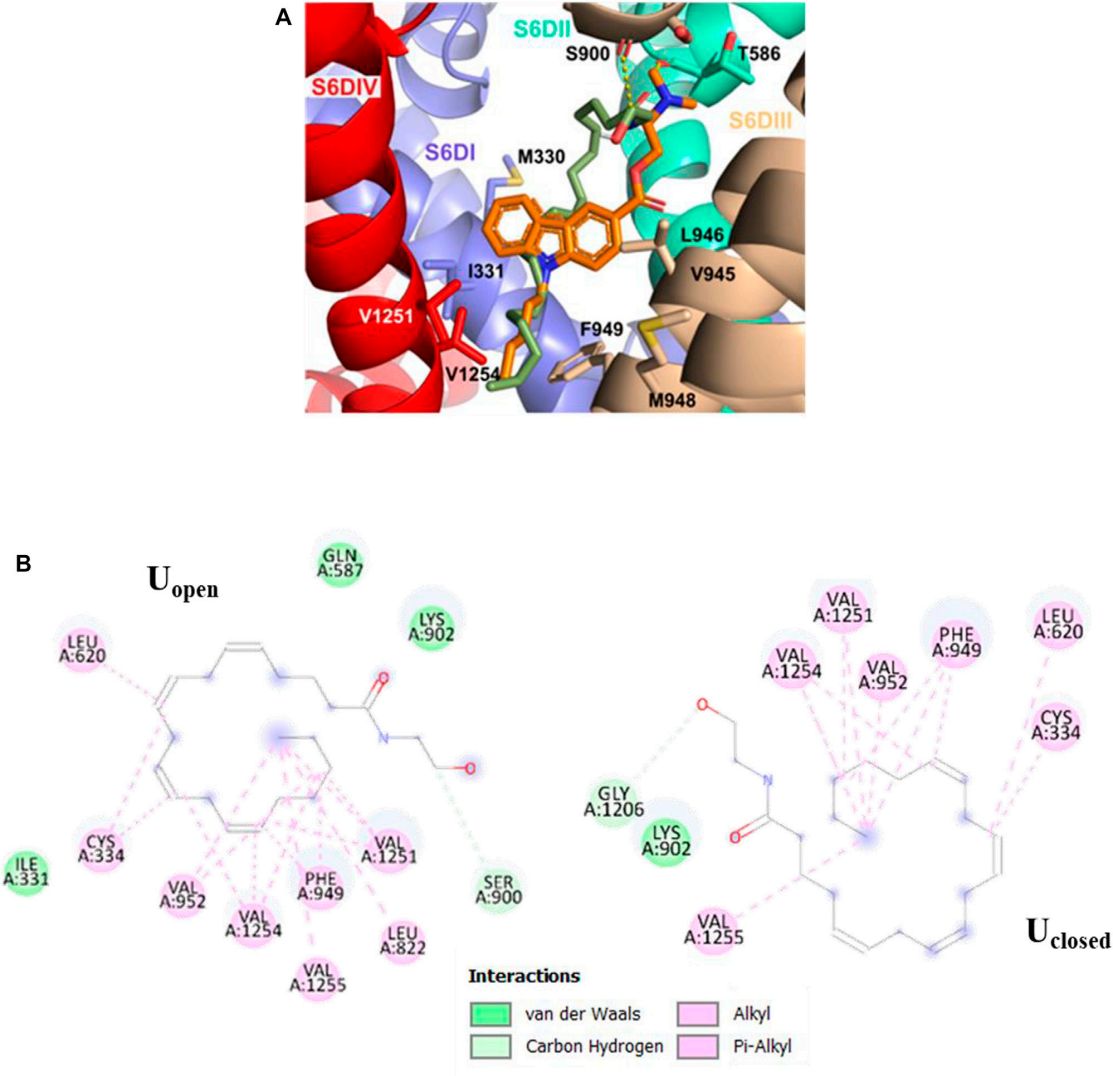
Molecular interactions of U_closed_, U_open_ (green), and NMP-181 (orange) with Ca_v_3.2. **(A)** 3D perspective of U_open_ and NMP-181 at the pore-blocking site in the Ca_v_3.2 channel; **(B)** 2D perspective interaction for U_closed_ and U_open_ anandamide conformer in the pore-blocking site.

As for the pore-blocking site, we identified a common hydrophobic region formed by the residues V1251, V1254, M948, F949, V945, L946, I331, and M330, where the non-polar tail (Alkyl-F) of the anandamide conformer U_open_ and the pentyl group of the NMP-181 compound established alkyl-alkyl interactions. In these molecular poses, the carbonyl oxygen of S900 and T568 residues in Ca_v_3.2, form a hydrogen bond with the polar head (C2’−H⋯O) of the conformer U_open_ of anandamide and the amine group (C7−H⋯O) of the NMP-181, respectively. The conformer U_closed,_ in the pore-blocking site, is located very similar to the conformer U_open_ (Figure 5) but the EA group is oriented towards the segment P of the DI forming a polar interaction (C−H⋯O22) with O22 of anandamide and the carbon of G1206. In this context, it can be observed an equivalence of the interaction modes of the functional groups of anandamide and the NMP ligands (Rangel-Galván et al., 2022b) with the channel Ca_v_3.2. Electrophysiological experimental registers show a blocking preference of the inactivation phase of the Ca_v_3.2 channel by both anandamide (Chemin et al., 2001) and the NMP compounds (You et al., 2011; Gadotti et al., 2013; Bladen et al., 2015) was the MFV residues, located in the site S6-DIII, contribute to the inactivation phase of the Ca_v_3 channels (Marksteiner et al., 2001). In the case of the Ca_v_3.2, the corresponding residues are M948, F949, and V950 which are located in the pore-blocking site. Also, in a recent study, the Ca_v_3.1/Z944 complex was solved by cryo-EM (Zhao et al., 2019), where common hydrophobic interacting residues stabilize the ligand binding pose, including the residue T921 (which is equivalent to T586 of the Ca_v_3.2 located in the pore-blocking site). This ligand binding site was proposed also for the genistein/Ca_v_3.3 complex using a combination of *in vitro* and *in silico* technics for the study (Rangel-Galván et al., 2021). Another recent study mapping the binding site of the Ca_v_3.1 channel with a small cyclic peptide PnCs1 using docking and molecular dynamics showed a pocket located mainly in the pore region and including some fenestration (Depuydt et al., 2021). Based on the aforementioned experimental evidence, it is possible that the most probable pose for this type of ligand is located in the pore-blocking site.

**FIGURE 6:**
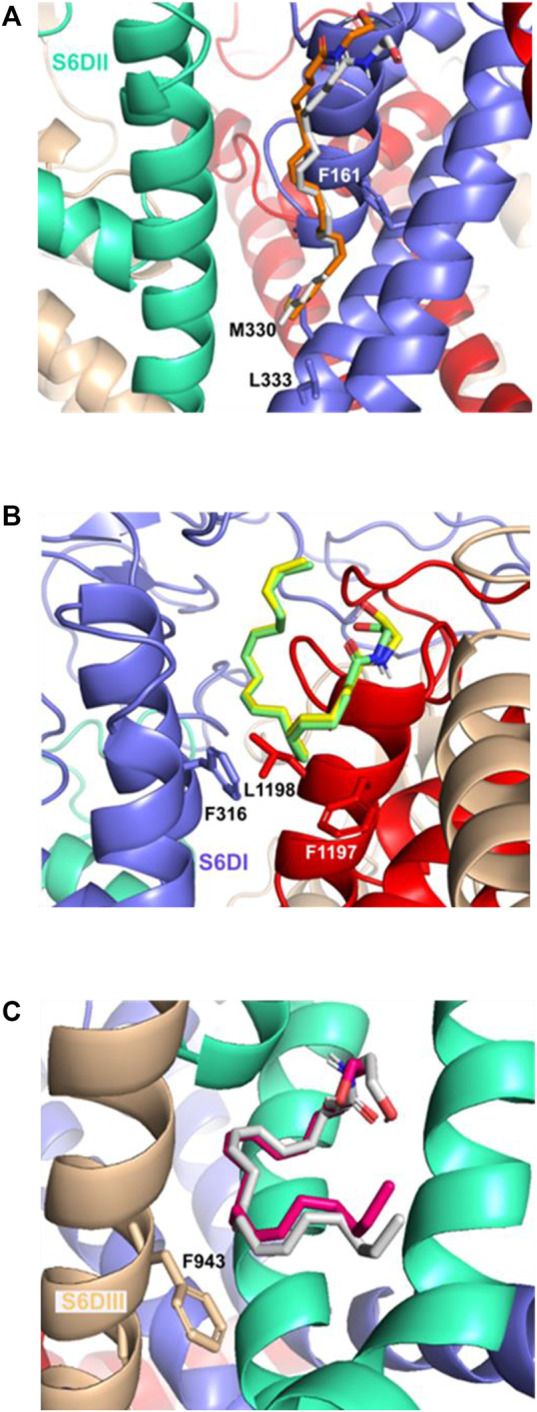
Important residues interaction of the Ca_v_3.2 channel and the anandamide conformers: **(A)** S6DII site and the E_closed_ and E_open_ conformer, **(B)** S6DI site and the H_closed_ and H_open_ conformers, **(C)** S6DIII site and the H_closed_ and H_open_ anandamide conformers with π interactions mainly with the F943 residue.

In the pose S6DII, the E_closed_ and E_open_ anandamide conformers (Figure 6A) form a π-σ interaction with residue F161 while the Alkyl-F region forms an alkyl interaction with residues M330 and L333. The H_closed_ and H_open_ conformers form a π−σ interaction with the residues F316 and F1197 in the S6DI site (Figure 6B) and with the F943 in the S6DIII site (Figure 6C).

The energy values are summarized in Table 4. According to this analysis, the higher interaction energy in the Ca_v_3.2/anandamide complex corresponds to the E_closed_ conformer in the S6DI site. Nevertheless, due to anandamide flexibility is more probable for anandamide to prefer folded conformers. Indeed, the X-ray data of anandamide interacting with its protein transporter (FABP5) show a hairpin shape conformer (Sanson et al., 2014) and according to MD simulations, anandamide prefers curved shapes in the pocket binding FAAH interaction (Palermo et al., 2013).

**TABLE 4:** Interaction energies ΔG (kcal mol^−1^) of the conformers of anandamide with the channel Ca_v_3.2.

	NMP-4^a^	NMP-7^a^	NMP-181^a^	E_closed_	E_open_	U_closed_	U_open_	H_closed_	H_open_
S6DI	−9.1	−10.0	−9.6	−9.9	−9.2	−9.2	−8.8	−8.9	−8.3
S6DII	−9.5	−10.0	−9.1	−9.2	−8.8	−9.8	−9.8	−9.4	−9.4
S6DIII	−9.5	−10.1	−9.2	−8.5	−8.7	−8.8	−9.2	−8.6	−8.6
S5DIV	−7.9	−8.1	−8.2	−8.4	−8.3	−9.2	−9.2	−8.4	−8.6
Pore-blocking	−9.4	−9.1	−8.2	−8.9	−9.2	−8.7	−8.3	−8.4	−8.2

a From Rangel-Galván et al. (2022b).

It is observed that there is a negligible difference in the values for the interaction energy related to the formation of a seven-atom ring in the EA group (open versus closed conformations). Only in the S6DI site, a preference for the closed ring conformers is obtained with a difference not greater than 0.7 kcal mol^−1^. In addition, our results indicate that better interaction energy for the U-shape conformers (U_closed_/U_open_) with respect to the hairpin shape conformers (H_closed_/H_open_) is observed. Along these lines, the drug Z944 is placed in a U-shape form in the binding pocket of the T-type calcium channel (Zhao et al., 2019).

## Conclusion

Using conceptual DFT and QTAIM approaches with the BP86/cc-pVTZ level of theory, the structural stability and chemical reactivity properties of six preferred conformers of the endocannabinoid anandamide were analyzed. Our results indicate slight differences for U and H shapes relative to extended forms, in the global reactivity descriptors, concluding an increase in hardness, and a decrease in softness and the electrophilicity index. From these parameters, a decrease in the electrophilicity index was observed in the case of the open ring conformers with respect to their corresponding closed ring counterparts. Also, the hardness and the electrophilicity indexes change when the values for anandamide are compared with those of the NMP compounds. According to the local reactivity descriptors (Fukui and Parr functions), the most probable locations for electrophilic and nucleophilic attacks are located in the ethanolamide group and in the Alkyl-M region for all the anandamide conformers considered in this study. Molecular docking analysis showed important π-σ interactions of anandamide with the side chain of phenylalanine residues. From our results, we observed that, despite their marked structural differences, the NMP compounds (NMP-4, NMP-7, and NMP-181) and anandamide may share a similar physiological activity profile regarding ligand/receptor interactions. The carbazole group is an important region of electronic density accumulation for the NMP compounds in the same way that the Alkyl-M region is for anandamide. According to the literature and the results obtained, the pore-blocking site of the Ca_v_3.2 calcium channel could be a probable binding site for the anandamide molecule.

## Data Availability

The original contributions presented in the study are included in the article/Supplementary Material; further inquiries can be directed to the corresponding authors.
