# Supplementary material for: Conceptual DFT, QTAIM, and Molecular Docking Approaches to Characterize the T-Type Calcium Channel Blocker Anandamide
Source: Front Chem. 2022 Jul 14;10:920661. doi: 10.3389/fchem.2022.920661 (PMC9329692; doi:10.3389/fchem.2022.920661)
Supplement: Supplementary file 1 [file DataSheet1.docx]

Supplementary Material

**Conceptual DFT, QTAIM, and Molecular Docking Approaches to Characterize the T-type Calcium Channel Blocker Anandamide**

**Maricruz Rangel-Galván^1^, María Eugenia Castro^2*^, Jose Manuel Perez-Aguilar ^1^, Norma A. Caballero ^3^, Francisco J. Meléndez ^1*^**

^1^ Lab. de Química Teórica, Centro de Investigación, Depto. de Fisicoquímica, Facultad de Ciencias Químicas, Benemérita Universidad Autónoma de Puebla, Edif. FCQ10, 22 Sur y San Claudio, Ciudad Universitaria, Col. San Manuel, C.P 72570. Puebla, Puebla, México.

^2^ Centro de Química, Instituto de Ciencias, Benemérita Universidad Autónoma de Puebla, Complejo de Ciencias, ICUAP, Edif. IC8, 22 Sur y San Claudio, Ciudad Universitaria, Col. San Manuel, C.P 72570. Puebla, Puebla, México.

^3^ Facultad de Ciencias Biológicas, Benemérita Universidad Autónoma de Puebla, Edif. BIO1, 22 Sur y San Claudio, Ciudad Universitaria, Col. San Manuel, C.P 72570. Puebla, Puebla, México.

*** Correspondence:**María Eugenia Castro
mareug.castro@correo.buap.mx

Francisco J. Melendez
francisco.melendez@correo.buap.mx

**Content**

**Table S1** Fukui functions, $f^{+}\left( r \right)$ and $f^{-}\left( r \right)$, dual descriptor, $f^{\left( 2 \right)}\left( r \right)$, and Parr functions, $P^{-}\left( r \right)$ and $P^{+}\left( r \right)$ of the conformers of anandamide: E_open_, U_open_, and H_open_, obtained at the BP86/cc-pVTZ level of theory in chloroform.

**Figure S1** Fukui functions, *f ^+^* and *f ^–^*, of the conformers of ananadamide: E_open_, U_open_, and H_open_, obtained at the BP86/cc-pVTZ level of theory in chloroform.

**Table S2.** Topological parameters (a.u.), *E_H…Y_* (kcal mol^-1^) and interatomic distances (D_inter_, Å) of the conformers of anandamide: E_open_, U_open_, and H_open_, obtained at the BP86/cc-pVTZ level of theory in chloroform.

**Figure S2** Molecular graphs of the conformers of anandamide: E_open_, U_open_, and H_open_, obtained at the BP86/cc-pVTZ level of theory in chloroform.

**Table S1** Fukui functions, $f^{+}\left( r \right)$ and $f^{-}\left( r \right)$, dual descriptor, $f^{\left( 2 \right)}\left( r \right)$, and Parr functions, $P^{-}\left( r \right)$ and $P^{+}\left( r \right)$ of the conformers of anandamide: E_open_, U_open_, and H_open_, obtained at the BP86/cc-pVTZ level of theory in chloroform.

|  |  | $f^{+}\left( r \right)$ | $f^{-}\left( r \right)$ | $f^{\left( 2 \right)}\left( r \right)$ | $P^{-}\left( r \right)$ | $P^{+}\left( r \right)$ |
| --- | --- | --- | --- | --- | --- | --- |
| E_open_ | C1 | 0.020 | 0.022 | 0.002 | 0.015 | 0.046 |
|  | C5 | 0.048 | 0.051 | 0.003 | 0.076 | 0.078 |
|  | C6 | 0.042 | 0.043 | 0.001 | 0.071 | 0.074 |
|  | C8 | 0.045 | 0.059 | 0.014 | 0.071 | 0.094 |
|  | C9 | 0.043 | 0.057 | 0.013 | 0.068 | 0.093 |
|  | C11 | 0.044 | 0.054 | 0.010 | 0.070 | 0.090 |
|  | C12 | 0.046 | 0.059 | 0.013 | 0.073 | 0.092 |
|  | C14 | 0.046 | 0.034 | 0.012 | 0.081 | 0.058 |
|  | C15 | 0.054 | 0.045 | 0.009 | 0.088 | 0.070 |
|  | O21 | 0.079 | 0.023 | 0.056 | 0.145 | 0.022 |
| U_open_ | C1 | 0.023 | 0.017 | 0.005 | 0.015 | 0.035 |
|  | C5 | 0.040 | 0.044 | 0.004 | 0.063 | 0.065 |
|  | C6 | 0.042 | 0.038 | 0.004 | 0.074 | 0.069 |
|  | C8 | 0.036 | 0.055 | 0.019 | 0.055 | 0.088 |
|  | C9 | 0.032 | 0.050 | 0.018 | 0.051 | 0.079 |
|  | C11 | 0.051 | 0.062 | 0.011 | 0.085 | 0.104 |
|  | C12 | 0.050 | 0.064 | 0.014 | 0.084 | 0.103 |
|  | C14 | 0.049 | 0.042 | 0.007 | 0.090 | 0.075 |
|  | C15 | 0.055 | 0.050 | 0.005 | 0.087 | 0.076 |
|  | O21 | 0.087 | 0.020 | 0.067 | 0.152 | 0.017 |
| H_open_ | C1 | 0.022 | 0.028 | 0.006 | 0.016 | 0.059 |
|  | C5 | 0.043 | 0.037 | 0.006 | 0.073 | 0.045 |
|  | C6 | 0.042 | 0.033 | 0.008 | 0.079 | 0.077 |
|  | C8 | 0.028 | 0.059 | 0.031 | 0.037 | 0.091 |
|  | C9 | 0.019 | 0.050 | 0.032 | 0.023 | 0.084 |
|  | C11 | 0.046 | 0.055 | 0.009 | 0.064 | 0.090 |
|  | C12 | 0.053 | 0.060 | 0.007 | 0.096 | 0.096 |
|  | C14 | 0.041 | 0.036 | 0.005 | 0.076 | 0.059 |
|  | C15 | 0.043 | 0.045 | 0.002 | 0.059 | 0.071 |
|  | O21 | 0.089 | 0.029 | 0.061 | 0.163 | 0.028 |

##


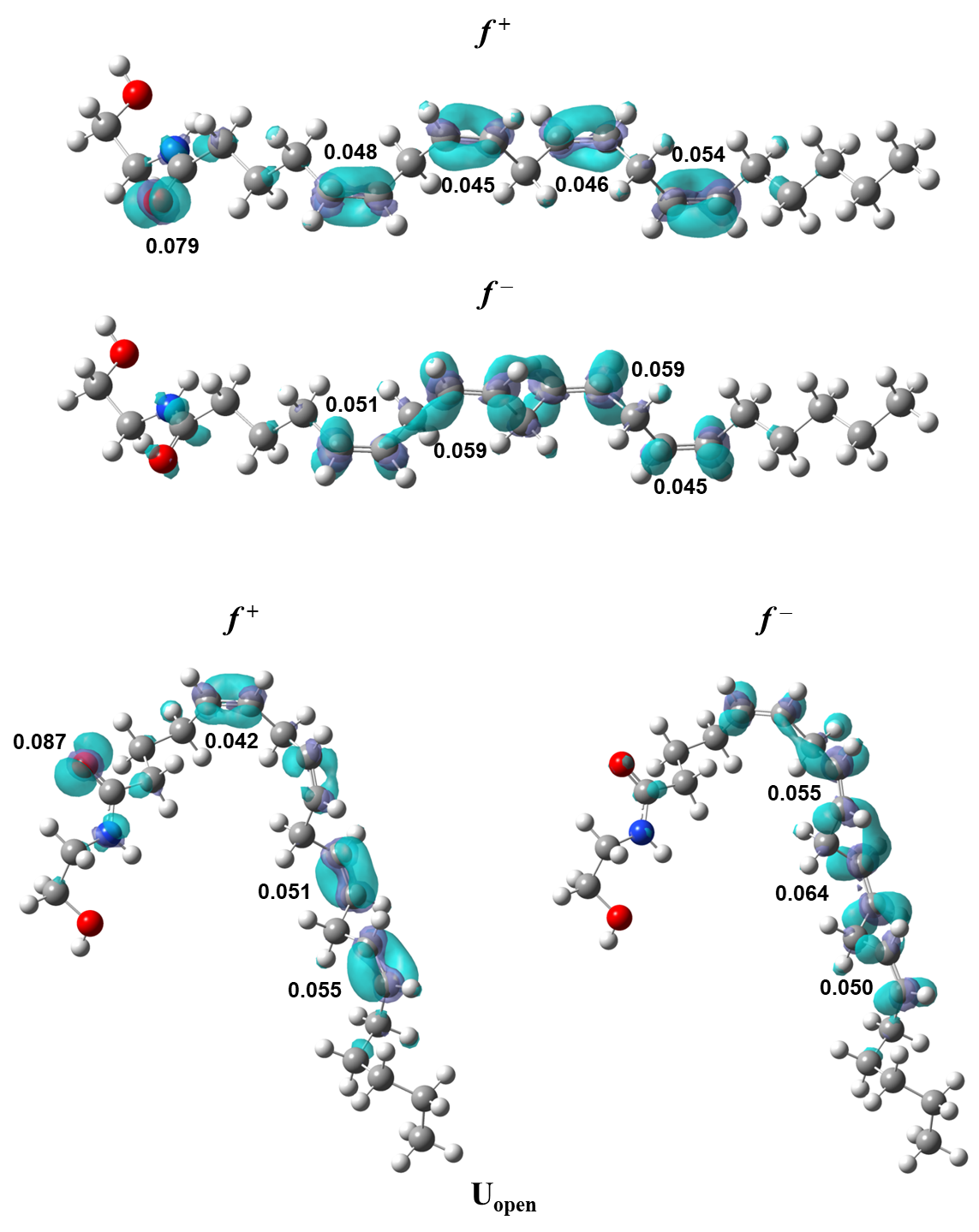


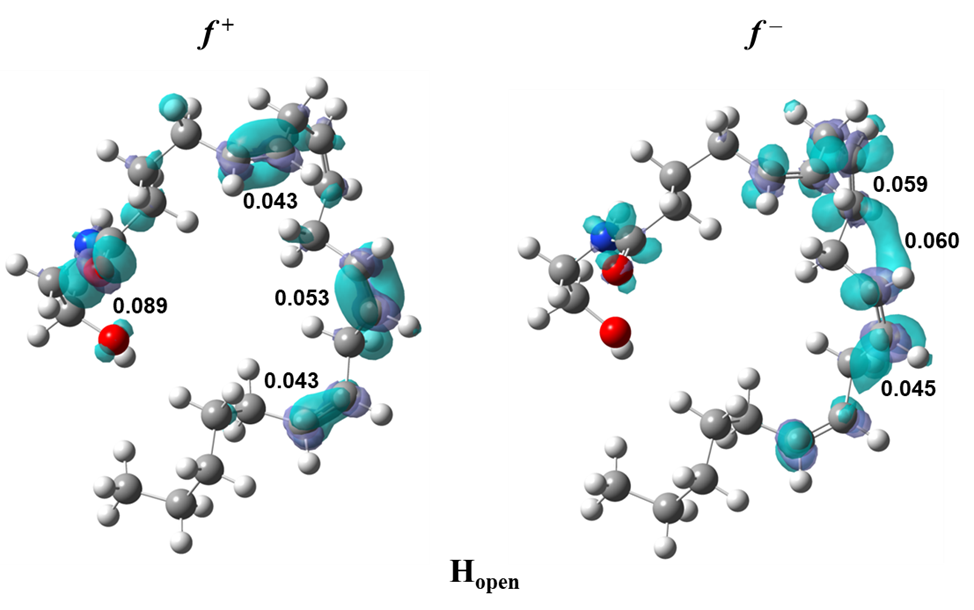


**Figure S1** Fukui functions, *f ^+^* and *f ^–^*, of the conformers of ananadamide: E_open_, U_open_, and H_open_, obtained at the BP86/cc-pVTZ level of theory in chloroform.

**Table S2.** Topological parameters (a.u.), *E_H…Y_* (kcal mol^-1^) and interatomic distances (D_inter_, Å) of the conformers of anandamide: E_open_, U_open_, and H_open_, obtained at the BP86/cc-pVTZ level of theory in chloroform.

| **BCP** | **ρ(r)** | **∇^2^ρ** | **G (r)** | **V (r)** | **H (r)** | **E_H…Y_** | **Dinter** | **DI** | **RCP** |
| --- | --- | --- | --- | --- | --- | --- | --- | --- | --- |
|  |  |  |  | **E_open_** |  |  |  |  |  |
| C_4_H_31_-H_35_C_7_ | 0.0118 | 0.0380 | 0.0079 | -0.0063 | 0.0016 | 1.98 | 2.06020 | 0.0285 | 0.0113 |
| C_7_H_34_-H_37_C_10_ | 0.0121 | 0.0387 | 0.0081 | -0.0065 | 0.0016 | 2.04 | 2.04347 | 0.0290 | 0.0115 |
| C_10_H_38_-H_42_C_13_ | 0.0121 | 0.0386 | 0.0081 | -0.0065 | 0.0016 | 2.04 | 2.04352 | 0.0291 | 0.0115 |
| C_13_H_41_-H_44_C_16_ | 0.0118 | 0.0379 | 0.0079 | -0.0063 | 0.0016 | 1.98 | 2.06105 | 0.0284 | 0.0113 |
|  |  |  |  | **U_open_** |  |  |  |  |  |
| C_4_H_31_-H_35_C_7_ | 0.0111 | 0.0366 | 0.0076 | -0.0060 | 0.0016 | 1.88 | 2.09088 | 0.0263 | 0.0008 |
| C_7_H_35_-H_37_C_10_ | 0.0116 | 0.0378 | 0.0079 | -0.0063 | 0.0016 | 1.98 | 2.06399 | 0.0272 | 0.0112 |
| C_10_H_38_-H_42_C_13_ | 0.0121 | 0.0385 | 0.0081 | -0.0065 | 0.0016 | 2.04 | 2.04259 | 0.0292 | 0.0115 |
| C_13_H_41_-H_44_C_16_ | 0.0119 | 0.0384 | 0.0080 | -0.0064 | 0.0016 | 2.01 | 2.05883 | 0.0284 | 0.0114 |
|  |  |  |  | **H_open_** |  |  |  |  |  |
| C_4_H_31_-H_35_C_7_ | 0.0121 | 0.0383 | 0.008 | -0.0065 | 0.0015 | 2.04 | 2.04707 | 0.0293 | 0.0115 |
| C_6_-H_38_C_10_ | 0.0133 | 0.0389 | 0.0085 | -0.0073 | 0.0012 | 2.29 | 2.32425 | 0.0448 | 0.0092 |
| C_10_H_37_-H_42_C_13_ | 0.0116 | 0.0377 | 0.0078 | -0.0063 | 0.0015 | 1.98 | 2.06453 | 0.0272 | 0.0111 |
| C_13_H_42_-H_44_C_16_ | 0.0116 | 0.0377 | 0.0078 | -0.0063 | 0.0015 | 1.98 | 2.06636 | 0.0274 | 0.0112 |
| C_17_H_45_-O_22_ | 0.0022 | 0.0072 | 0.0014 | -0.001 | 0.0004 | 0.31 | 3.18881 | 0.0098 | 0.0018 |
| C_20_H_53_-O_22_ | 0.0024 | 0.0071 | 0.0014 | -0.0011 | 0.0003 | 0.35 | 3.18106 | 0.0095 | 0.0018 |


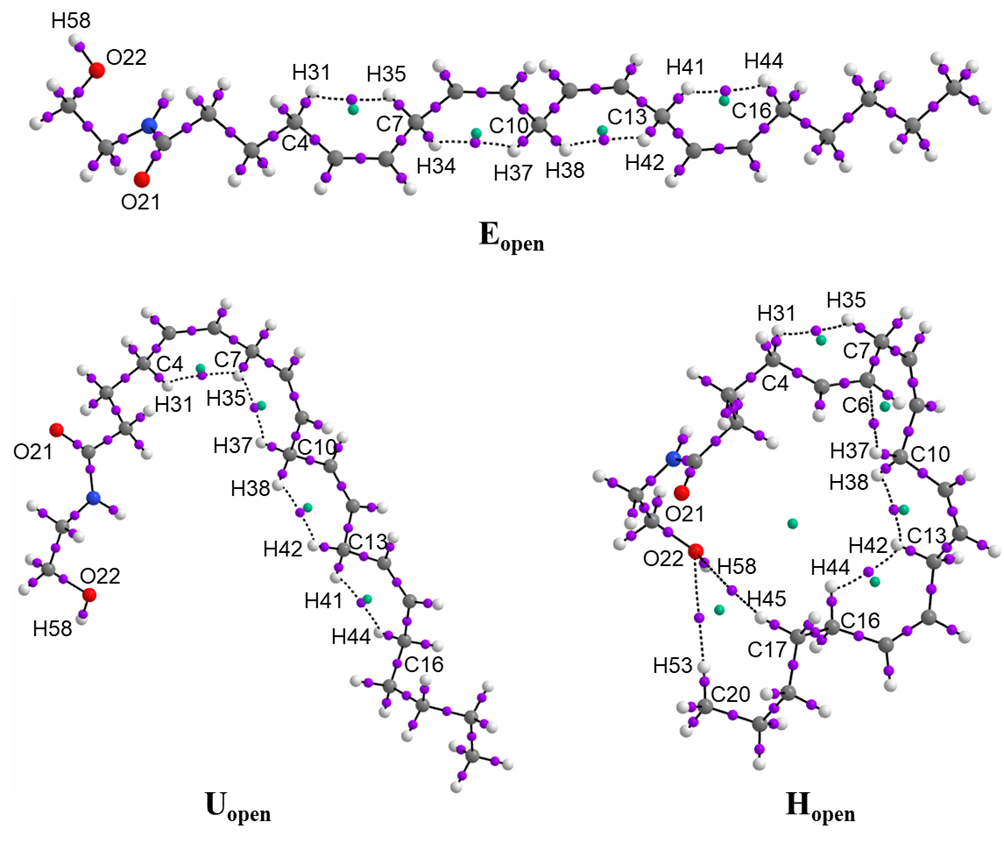


**Figure S2** Molecular graphs of the conformers of anandamide: E_open_, U_open_, and H_open_, obtained at the BP86/cc-pVTZ level of theory in chloroform.
